# Morphological and growth altering effects of Cisplatin in *C. albicans *using fluorescence microscopy

**DOI:** 10.1186/1476-0711-4-7

**Published:** 2005-04-25

**Authors:** Chandrasekhar Kesavan, Malathi Raghunathan, Natarajan Ganesan

**Affiliations:** 1 Musculoskeletal Disease Center, JLP VA Medical Center, Loma Linda, CA 92354, USA; 2 Department of Genetics, Dr. ALM Post Graduate Institute of Basic Medical Sciences, Chennai-600 113, India; 3 240 Reiss Science Bldg. Georgetown University, 37th & O Sts NW, Washington DC – 20057, USA

## Abstract

Changes in morphology and growth curve of *Candida albicans *in response to treatment by Cisplatin has been studied using fluorescence staining with ethidium bromide. Treatment with Cisplatin was found to markedly inhibit hyphae and ovoid growth as revealed by ethidium bromide staining of drug treated cells. These changes were concomitant with inhibitory effects on the growth curve with respect to untreated cells

Presence of Cisplatin not only caused suppression in the limiting values in the growth curve, but also caused a slight left shift in the EC50 values. Some of the ovoid cells undergoing poisoning with cisplatin were found to be unusually enlarged before undergoing their natural fate thus suggesting formation of similar cytotoxic end products with DNA.

## Introduction

*Candida albicans *is a yeast pathogen that causes mild, chronic, superficial systemic infections in immunocompromised and cancer patients [1-3]. Research, worldwide is broadly focused on 1) search of new targets in *C. alibcans*, 2) Screening inhibitory activities with therapeutic agents, and 3) designing or modifying novel agents, to overcome treatment resistance [4]. *C. alibcans *has been shown to harbor a self splicing group-I intron in the nuclear 25s rRNA genes [5]. Presence of this gene in *C. alibcans*, but not in the human genome, suggests that splicing inhibitors could potentially act as alternate targets in controlling the proliferation of fungal pathogens. Recent screening studies using disk diffusion have shown that cis-diamminedichloro platinum (Cisplatin), a well-established antitumor agent, inhibits the growth of *C. alibcans *at lower concentrations [6]. Despite its prevalent use in cancer treatment, very little work has been done [7-10] to document the effects of Cisplatin and related metal complexes in controlling the proliferation behavior of *C. alibcans*. With the molecular mechanisms remaining largely unknown, this provided us a basis to study the effects of Cisplatin treatment on the morphology and growth curve of *C. alibcans*. Morphological changes in *Candida *can be followed by direct fluorescence staining with suitable dyes to selectively visualize different parts/aspects of cellular activities [11-13]. Though the use of Ethidium Bromide for fluorescence staining is well known, there is no report to show the use of this technique in studying the morphology of *Candida*, especially in response to treatment with anticancer agents such as Cisplatin. Herein, we report the morphological changes of *C. alibcans *in response to cisplatin using fluorescence microscopy with ethidium bromide staining.

## Materials and methods

### Media and Inoculums

Yeast isolates (clinical strains) were obtained from patients, Royapettah Hospital, Chennai. Sub-cultured isolates were maintained in Sabouraud dextrose agar (B.B.L, Cockeysville, MD) at 1–5 × 10^6 ^CFU/10 ml media. 30 μl of each sample (log phase) was used for slide preparation to study the morphological changes.

### Drug preparation

A 2 mg/ml stock of Cisplatin (TNDPL, Chennai) was prepared in sterile Millipore water. Stock was diluted to 60 μg/10 ml culture for treatment purposes.

### Staining

Ethidium bromide staining was performed using a freshly prepared stock of ethidium bromide (1 mg/ml water) from which 50 μl/100 ml concentration was used for staining the slides. Gram staining was performed from commercially available kits as per instructions.

### Morphological studies using Fluorescence Microscopy

30 μl of cultured *C. alibcans *(Drug treated/non-treated cells) was taken and spread over the clean glass slide and dried at 37°C for 5 minutes. Slides were dipped into the EtBr staining solution for 30–40 seconds followed by immersion in water for 15–20 seconds to remove excess stain. Gram staining was performed in parallel on a similar set of slides to validate the results. Both the set of slides were viewed using NFM under the 100× magnification.

## Results and Discussion

**Figure 1:**
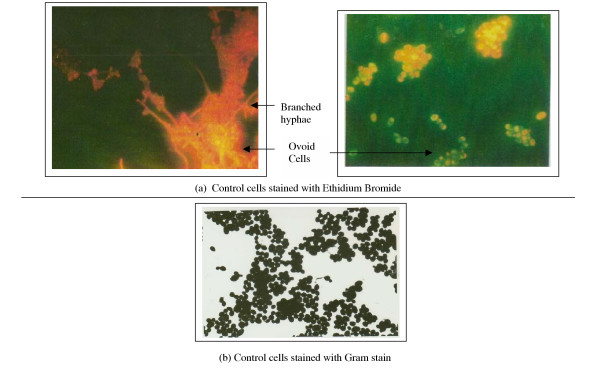
Photograph showing morphology of *Candida albicans *in the absence of cisplatin (6 ug/ml), stained with ethidium bromide and gram stain.

**Figure 2:**
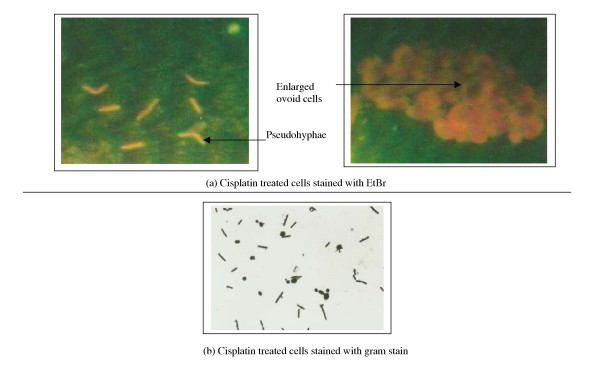
Photograph showing morphology of *Candida albicans *in the presence or absence of cisplatin (6 ug/ml), stained with ethidium bromide and gram stain.

**Figure 3:**
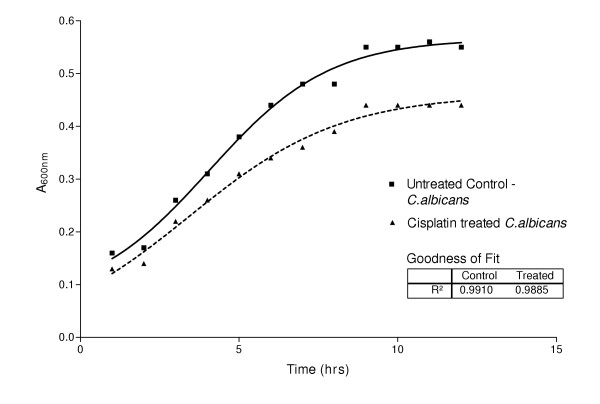
Growth curve of *Candida albicans *in the presence/absence of Cisplatin (6 μg/ml). Curve fitting of data points was performed using non-linear regression(sigmoidal curve): GraphPad Prism Version 3.02.

Treatment of *C. alibcans *with Cisplatin was found to markedly inhibit hyphae and ovoid growth (Fig. 2) as revealed by ethidium bromide staining of drug treated cells. This was in contrast to the controls showing markedly branched hyphae, with budding characteristics (Fig. 1). The ovoid cells of Cisplatin treated *C. alibcans *showed an increased uptake of the ethidium bromide stain (red color) in contrast to the untreated counterparts (yellowish orange), thus pointing to drug induced poisoning and death of treated cells. These changes were concomitant with inhibitory effects on the growth curve with respect to untreated cells (Fig. 3). Presence of Cisplatin not only caused suppression in the limiting growth values but caused a slight left shift in the EC50 values, which remains to be studied further. Some of the ovoid cells undergoing poisoning with cisplatin were found to be unusually enlarged before undergoing their natural fate (Fig. 2). This is known to be one of the typical effects of poisoning with cisplatin in case of tumor cell lines as well thus suggesting formation of similar cytotoxic end products [14]. Cisplatin in water forms a diaqua species of Pt^+2 ^due to the fast leaving Cl^- ^ions. This could lead further to host of reactions in the cytoplasm before forming end products with DNA. It is also possible that cisplatin exerts its activity through other secondary response mechanisms. Further studies using RT-PCR will elucidate 1) the role of the binding affinity of cis-DDP to certain RNA species in cytotoxicity against *C. albicans*, for example, group-I intron ribozyme, known in many human diseases causing pathogens and a potential target site for therapeutic agents. 2) Whether cis-DDP inhibits the *C. alibcans *growth through penetration into the cell or some secondary response.
